# Sudden Unexpected Death in Epilepsy: A Narrative Review of Mechanism, Risks, and Prevention

**DOI:** 10.3390/jcm14103329

**Published:** 2025-05-10

**Authors:** Rena Y. Jiang, Robin T. Varughese, Sanjeev V. Kothare

**Affiliations:** 1 College of Medicine, University of South Florida Morsani, Tampa, FL 33602, USA; jiang.rena.030@gmail.com; 2 Division of Pediatric Neurology, Cohen Children’s Medical Center, Northwell, New Hyde Park, NY 11042, USA; skothare@northwell.edu

**Keywords:** sudden unexpected death in epilepsy, seizure, generalized tonic clonic seizure, Dravet syndrome, channelopathy, postictal generalized EEG suppression, sleep disorder, heart rate variability, central apnea

## Abstract

Sudden unexpected death in epilepsy (SUDEP) is sudden, unexpected, witnessed or unwitnessed, nontraumatic, non-drowning death that occurs in a person with epilepsy. SUDEP is the leading cause of epilepsy-related death in adults with epilepsy, with an incidence of about 1.2 per 1000 person-years in the general epilepsy population. Recent studies have shown similar prevalence in the pediatric population too. Although the precise mechanism remains unclear, well-documented cases of SUDEP suggest that a generalized tonic clonic seizure-induced, centrally mediated change in cardiorespiratory function leads to terminal apnea and cardiac arrest. Risk factors include generalized tonic clonic seizure frequency, duration of epilepsy, nocturnal seizure, and certain genetic syndromes. Orexin, adenosine, and serotonin neurotransmission have been explored as novel drug targets to mitigate SUDEP risk. Neurostimulation and resective epilepsy surgery have been reported to have beneficial effects on long-term SUDEP risk as well. Future studies may aim to clarify the role of sleep and other comorbidities in SUDEP pathophysiology.

## 1. Introduction

Epilepsy, a neurological condition characterized by recurrent unprovoked seizures, affects millions of people across the world [1]. Sudden unexpected death in epilepsy (SUDEP) is the leading cause of epilepsy-related death in adults with epilepsy [2,3]. SUDEP can be described as sudden, unexpected, witnessed or unwitnessed, nontraumatic, non-drowning death that occurs in a person with epilepsy. SUDEP should occur in benign circumstances, and it can occur with or without evidence of a seizure. Documented status epilepticus should be excluded, as this can be a cause of death per se. Furthermore, post-mortem examinations should not reveal an alternative cause of death [4,5]. A classification of near, possible, probably, and definite SUDEP can be performed using the criteria described by Nashef et al., 2012 [4] (Table 1). The following review will explore SUDEP epidemiology, its proposed mechanisms, its risk factors, its potential biomarkers, its mitigation measures, its counseling recommendations, and future directions.

## 2. Epidemiology

SUDEP can occur in both pediatric and adult epilepsy patients. The incidence in person-years for adults with epilepsy is about 1.2 per 1000 [6,7,8]. The incidence appears to be lower in younger adults, at 1.13, and higher in adults between 20 and 40 years old, at 1.29 per 1000 person-years [7]. The incidence is higher in drug-resistant epilepsy, at about 6.7 per 1000 [9]. Previously, SUDEP was thought to be relatively uncommon in pediatric epilepsy patients compared to adults, at about 0.22 per 1000 person-years [6,10]. However, relying only on death certificates may underestimate SUDEP incidence. Studies from Sweden [7] and Canada [11] have shown that the incidence of SUDEP in pediatric patients may be similar to that of adults, at about 1.11 to 1.17 per 1000 person-years. It is thought that potential differences in SUDEP incidence between pediatric and adult populations may be related to differences in the mechanistic underpinnings [12], but there remains a lack of studies in the pediatric space to fully understand the pathophysiology.

Low socioeconomic status has been associated with higher SUDEP incidence. Over a five-year period between 2010 and 2015, the SUDEP rate ratio between the lowest and highest socioeconomic status quartiles increased from 2.6 to 3.3, despite decreased overall SUDEP rates [13].

While SUDEP is more likely to occur in the setting of refractory epilepsy, it can still occur in treatment-responsive or benign epilepsy [14]. SUDEP has also been reported in self-limited epilepsy with centrotemporal spikes [15].

## 3. Mechanism

Our mechanistic understanding of SUDEP is mostly based on adult studies and preclinical animal models (Table 2). In adults, SUDEP is thought to be the result of centrally mediated changes in cardiorespiratory function following, in most cases, a generalized tonic clonic seizure (GTCS) [16]. However, convincing cases of SUDEP have been reported without preceding seizure in patients with intractable epilepsy and frequent GTCS [17]. In these cases, the mechanism remains unclear, although a primary brainstem mechanism affecting respiration is suspected [17]. The brain structures involved in SUDEP pathogenesis include limbic, paralimbic, and brainstem structures including the amygdala, hippocampus, periaqueductal gray, pons, medulla, and raphe nuclei [18,19].

### 3.1. Respiratory

There are several components of respiratory compromise that ultimately contribute to SUDEP. During generalized and focal seizures, respiratory changes can include central apnea, obstructive events, desaturation, hypercapnia with acidosis, bradypnea, and tachypnea [20]. Ictal central apnea is more prevalent in patients of a younger age, with left temporal lobe onset seizures, longer duration of seizure, and patients taking more antiseizure medications [21,22]. Ictal central apnea is driven by limbic and paralimbic structures involved in the mesial temporal breathing modulation network, which includes the amygdala, hippocampus, and anterior parahippocampal and antero-mesial fusiform gyri [23,24,25]. Damage to these areas has been seen in patients at high risk for SUDEP or who have experienced SUDEP [23]. Neuropathological studies of SUDEP have shown damage in chemosensing and brainstem respiratory control structures such as raphe nuclei, periaqueductal gray, and the medial posterior thalamus [26,27]. Poor chemosensing of carbon dioxide may lead to inadequate respiratory control, ultimately resulting in SUDEP. Functional magnetic resonance imaging (MRI) studies have shown that people with epilepsy have greater serotonergic modulation in the dorsal raphe compared to healthy controls [28]. During hypercapnia, people with epilepsy appear to recruit more structures for chemosensing and respiratory control [28]. Preclinical studies in KCNA1 null mice, which represent a temporal lobe epilepsy and SUDEP model, show progressive respiratory dysfunction and eventual SUDEP [29]. Methacholine-provoked hyperventilation attenuates with age, which increases the risk of SUDEP over time [29]. This model also appears to have hyperventilation-induced apnea, rather than hyperventilation followed by arousal, which suggests chemosensory instability.

Neurogenic pulmonary edema can occur with SUDEP, likely due to a seizure-provoked sympathetic surge that leads to generalized vasoconstriction and increased pulmonary vascular pressure [3,30,31]. Pulmonary edema may lead to ictal desaturation and hypercapnia independent of ictal apnea, and the resulting acidosis can then predispose patients to cardiac arrhythmia and downstream mechanisms of SUDEP [32].

In addition, postictal laryngospasm that occurs due to tonic discharges in bulbar nuclei can also contribute to respiratory compromise [20,33]. About 69% of SUDEP occurs during sleep [34], and 73.3% of SUDEP occurs in a prone position [35]. Asphyxiation while being unable to move from a prone position likely plays a role in SUDEP as well.

Preclinical studies have supported the neurovascular hypothesis of SUDEP, which emphasizes postictal vasoconstriction with subsequent hypoperfusion and hypoxia as the driving force behind SUDEP [36]. The hippocampus and pre-Bötzinger complex in the brainstem are involved in breathing rhythmogenesis. In animal models of SUDEP, partial pressure of oxygen is seen to drop in these regions after seizure [36]. In both acute and chronic mouse models of SUDEP, cyclooxygenase-2 inhibitor and L-type calcium channel antagonists successfully blocked seizure-induced vasoconstriction, thus reducing postictal hypoxia and extending their lifespan [36].

### 3.2. Cardiac

Seizures can lead to a variety of cardiac abnormalities including heart rate variability (HRV), tachycardia, bradycardia, sinus arrest, QT interval shortening, QT interval prolongation, and atrial fibrillation [37,38,39,40,41,42]. Temporal lobe epilepsy is associated with increased risk for asystole [43]. This can cause death in patients who are not otherwise considered high risk for SUDEP based on other elements of their history, such as epilepsy duration and occurrence of GTCS.

In general, ictal bradycardia occurs more in pediatric patients compared to adults, whereas ictal tachycardia occurs more in adults [22]. However, ictal bradycardia is more prevalent in patients of an older age with right hemispheric-onset seizures [21].

Sudden cardiac death (SCD), while not the same entity as SUDEP, shares several pathophysiologic features with SUDEP. Up to 82% of people with epilepsy have cardiovascular comorbidities [41]. Patients usually have associated cardiac conditions such as coronary artery disease, myocardial infarction, myocardial fibrosis, left ventricular hypertrophy, or conduction abnormalities [44]. SCD incidence in people with chronic epilepsy is about three-fold greater than that in the general population [45]. SCD usually occurs during daily activities, rather than during sleep like SUDEP does [41]. Two thirds of SCD are not associated with seizures. Similar to the SUDEP patient population, SCD patients may develop acquired channelopathies contributing to death. Autonomic modulation is related to both conditions as well. This can lead to an imbalance in parasympathetic–sympathetic drive and cardiac electrical instability [41].

### 3.3. Postictal Generalized Electroencephalogram Suppression (PGES)

Postictal generalized electroencephalogram (EEG) suppression (PGES) is a phenomenon characterized by an EEG amplitude less than 10 µV in the immediate postictal period [46,47]. It occurs in 27–82% of GTCS but only rarely in focal seizures [48]. There is no relation between the duration of the GTCS and the occurrence or the length of PGES [46,49,50]. Adults have a higher prevalence of PGES with longer durations compared to pediatric populations [22,51]. PGES occurs at the end of the tonic phase of a seizure. The length of the tonic phase, during which breathing is withheld, does correlate with the subsequent occurrence of PGES [52,53,54]. The magnitude of autonomic changes in response to GTCS corresponds to the duration of PGES [49]. This window of PGES and postictal autonomic changes, which include tachycardia, bradycardia, QT prolongation, and heart rate variability, could suggest a critical window of vulnerability for SUDEP pathogenesis. Given the same PGES duration, pediatric patients mount a stronger sympathetic response compared to adults [55]. This may explain why people become more vulnerable to SUDEP as they age.

### 3.4. Neurotransmitters

Adenosine appears to play an important role in SUDEP pathogenesis [56]. Adenosine is released in the setting of a seizure due to high energy expenditure in the form of adenosine triphosphate. Studies have shown that adenosine acts as an anticonvulsant and a seizure abortive via presynaptic and postsynaptic mechanisms [57,58]. However, adenosine acts as a double-edged sword in that it inhibits respiration in the postictal period via medullary pathways [53,59,60,61,62]. This can contribute to the cascade of events that eventually lead to terminal apnea and cardiac arrest in SUDEP. Furthermore, adenosine may contribute to PGES and sleep alterations, which are also involved in the pathophysiology of SUDEP [61].

Serotonergic pathways in the raphe nuclei and medulla play a key role in reticular formation, which is important for arousal and autonomic function [33]. Selective serotonin reuptake inhibitors (SSRIs) have been shown to decrease hemoglobin desaturation in refractory focal epilepsy [63], and fenfluramine enhances serotonin release to prevent respiratory arrest in a mouse model of SUDEP [64]. The action of serotonin on the periaqueductal gray helps restore respiration in response to hypercapnia in the setting of seizure-induced respiratory depression [56]. Clearly, serotonin plays an important role in oxygen stability in the setting of seizure and SUDEP. Serotonergic neurons also appear to raise the seizure threshold and decrease seizure mortality in preclinical models that mimic features of SUDEP [65]. Higher interictal serotonin levels are associated with shorter PGES [66], which reinforces the role of serotonin in postictal recovery.

In pediatric patients, there appears to be parallels between SUDEP and sudden infant death syndrome (SIDS). SIDS is the sudden unexplained death of infants under 12 months of age. The pathophysiology of SIDS can be understood using the “triple risk model,” which proposes that intrinsic vulnerability met with an exogenous trigger during a critical development period leads to SIDS [67]. In 50–75% of SIDS, there is abnormal serotonergic signaling in the brainstem [33,68,69]. Furthermore, about 40% of SIDS cases had hippocampal disorganization, which can lead to impairment of respiratory regulation even before the clinical onset of a seizure [70]. This parallels the neuropathological and imaging findings seen in SUDEP relating to respiratory control [28]. Prone positioning is a known risk factor for both SIDS and SUDEP, as it increases risk for asphyxiation [16,35,71].

### 3.5. Ferroptosis

Emerging evidence in preclinical studies suggests that iron accumulation may play a role in SUDEP [72,73,74]. While iron is important for many cellular processes, the dysregulation of iron can lead to a type of programmed cell death called ferroptosis. Ferroptosis involves processes including the generation of reactive oxygen species, lipid peroxidation, and cellular iron accumulation [74]. Similar processes are known to occur in the setting of epilepsy. Iron accumulation in cardiac tissue may contribute to some of the cardiac mechanisms involved in SUDEP pathogenesis [72,73,74].

## 4. Risk Factors

SUDEP-7 is a risk inventory based on factors identified in a study by Walczak et al. that reports individual SUDEP risk on a scale of 1 to 7 [9,75,76].

Risk factors for SUDEP include nocturnal seizures [77,78], early onset epilepsy [79], frequent GTCS [9,80], prolonged PGES [46], and genetic syndromes [2] (Table 3 and Table 4).

Nocturnal seizures are a risk factor for SUDEP [77,78]. The majority of SUDEP cases are sleep-related and often occur in the early morning hours of 4 am–8 am [78]. Interestingly, the incidence of sudden cardiac death and SIDS also appears to peak in the early morning hours [81,82]. Patients who experience sleep-related SUDEP are more likely to have a history of nocturnal seizures than those who experienced non-sleep-related SUDEP [78]. Nocturnal seizures are associated with more severe hypoxemia and PGES [77]. Seizures that occur during sleep lead to lower oxygen saturation compared to seizures that occur during wakefulness, both during ictal and postictal periods. PGES occurred in 39% of sleep seizures, compared to only 8% of wake seizures [77].

There is an increased mortality associated with early onset epilepsy due to SUDEP [83] as well as other epileptic and non-epileptic etiologies. A prospective study spanned over 40 years of 245 children with epilepsy diagnosed in 1964 showed a three-fold increased mortality in patients with childhood epilepsy compared to the general population [79]. Thirty percent of the deaths in this study were attributed to SUDEP. There was an increased risk of death with history of status epilepticus [79].

A strong risk factor for SUDEP is GTCS frequency [80,84]. Many patients with frequent GTCS are, reasonably, on multiple antiseizure medications (ASMs). Initial studies showed that polytherapy and even specific ASMs such as lamotrigine and carbamazepine may be associated with increased SUDEP risk [9,84]. However, a subsequent study showed that ASMs such as carbamazepine, phenytoin, valproic acid, and lamotrigine were not associated with increased SUDEP risk when GTCS frequency was taken into consideration [80]. In choosing ASMs to treat genetic epilepsy syndromes, however, it is important to consider the mechanism of action. For example, ASMs that block sodium channels can be used to treat patients with gain-of-function sodium channel mutations but not those with loss-of-function [85].

Epilepsy surgery has been associated with decreased seizure frequency and decreased mortality over time, despite still maintaining a higher mortality than the general population [86,87]. Patients who achieved good seizure control after epilepsy surgery had a lower chance of premature death afterwards, which reinforces the idea that seizure frequency is an important factor in mortality [86].

Prolonged PGES may also represent a risk factor for SUDEP. Prolonged PGES, defined as longer than 50 s, identifies refractory epilepsy patients who are at increased risk for SUDEP [46]. For every one second increase in the duration of PGES, the odds of SUDEP were found to increase by a factor of 1.7% [46].

There are a variety of genetic syndromes, including many channelopathies, that increase the risk for SUDEP [2,88,89]. Dravet syndrome is a severe infantile onset developmental and epileptic encephalopathy that can manifest with multiple seizure types, status epilepticus, intellectual disability, ataxia, and other clinical symptoms [89,90]. Most cases of Dravet syndrome are caused by a mutation in the SCNA1 gene, which encodes sodium voltage-gated channel alpha subunit 1 [89]. The rate of SUDEP in patients with Dravet syndrome is 9.32 per 1000 person-years, which is eight-fold higher than that of the general adult epilepsy population and still significantly higher than that of the refractory epilepsy population [89]. SUDEP in Dravet syndrome occurs mainly in childhood or adolescence [89].

Long QT syndromes (LQTSs) related to potassium and sodium channel mutations have been associated with SUDEP as well [2]. KCNQ1, which encodes the alpha subunit of a voltage-gated potassium channel, is a gene implicated in a common form of long QT syndrome. Preclinical studies show that KCNQ1 is expressed in both cardiac and brain tissue, specifically in forebrain neuronal networks and vagal brainstem nuclei [91]. The decreased ability to repolarize neurons via potassium channels after an action potential can be related to seizure and poor autonomic control in these forms of LQTS. Preclinical studies have shown that 62% of cardiac arrhythmias occurred with epileptiform discharges, which is consistent with the autonomic instability commonly associated with SUDEP [16]. Other LQTS genes associated with SUDEP include KCNH2, which encodes a component of a voltage-gated potassium channel, and SCN5A, which encodes a subunit of a voltage-gated sodium channel [2].

There is some evidence that acquired channelopathies may also contribute to SUDEP [88]. Preclinical studies have shown that epilepsy can alter the expression of cardiac ion channels such as voltage-gated sodium channels (Nav1.1, Nav1.5), voltage-gated potassium channels (Kv4.2, Kv4.3), sodium calcium exchangers (NCX1), and nonspecific conducting channels (HCN2, HCN4) [88].

Other genetic conditions that predispose patients to high seizure frequency and thus increase risk for SUDEP include 15q duplication syndrome [92], CDKL5 deficiency disorder [93], PCDH19-related epilepsy [94], and ring chromosome 20 syndrome [95].

## 5. Biomarkers

A biomarker is a feature that can be observed clinically and be used to anticipate or prevent a downstream catastrophic event such as SUDEP. In a survey of caregivers, about 85% of patients with SUDEP were reported to have multiple transient changes in health in the last few months leading up to death [96]. More than half of the patients had changes in seizures and sleep patterns. Smaller proportions of patients had changes in cognition, breathing rate, and heart rate. While this study may contain recall bias, it brings up the important idea that there may be subclinical features [14] that can help clinicians and caregivers implement interventions before the terminal event.

About one third of people with epilepsy have comorbid sleep disorders [97,98,99]. Often, epilepsy and sleep disorders appear to exacerbate each other [100]. Preclinical studies of epilepsy have demonstrated decreased non-rapid eye movement (NREM) and rapid eye movement (REM) sleep time, increased sleep fragmentation, and overall chronic sleep deprivation [101,102,103,104]. Recently, studies by Simeone, Iyer, and collaborators have begun to explore the role of orexin, a wake-promoting neuropeptide, in sleep and seizures in a SUDEP mouse model [29,100,105,106]. Kcna1 null mice are characterized by early onset epilepsy, interictal cardiac arrhythmias, sleep changes, and a high risk of SUDEP [107]. Studies of this mouse model have demonstrated an association between SUDEP risk and orexin levels in the lateral hypothalamus [105]. Previous studies have shown that orexin administration can promote seizures and blocking orexin receptors can reduce seizures [100,108,109,110]. Furthermore, kcna1 null mice mount an impaired ventilatory response to hypercapnia–hypoxia challenges [29,106] that mimic the respiratory pathophysiology seen in SUDEP [16]. An impaired central chemoreceptor system may underlie this problem: Kcna1 null mice have increased numbers of orexin neurons, which are responsive to pH and CO2 and important for modulating respiration [106]. The reason for elevated orexin levels in SUDEP is not clear. Chronic intermittent hypoxemia has been shown to increase prepro-orexin mRNA expression in rat hypothalamus [111]. Thus, orexin overexpression in kcna1 null mice may represent a compensatory mechanism to increase respiratory drive and stabilize blood gas [106]. High levels of orexin in the central nervous system may be further validated as a biomarker for SUDEP risk. The administration of a dual orexin receptor antagonist (DORA), which is a drug class approved for insomnia treatment in humans, increased survival in the SUDEP mouse model, improved sleep architecture, and improved the ventilatory response to hypercapnia–hypoxia challenges [29,105,106].

The association of HRV with SUDEP has been explored. Several studies have shown an inverse correlation of HRV, represented by root mean square differences (rMSD) of successive R-R intervals, and SUDEP risk in the general epilepsy population, as well as specifically patients with Dravet syndrome [39,42,75,112]. It appears that sodium channel mutations present in Dravet syndrome are associated with autonomic dysfunction, specifically unopposed sympathetic activity, that manifests as decreased HRV [42,112]. This could pose reduced HRV as a biomarker for SUDEP, but the subject requires more study.

Post-convulsive central apnea has also been associated with SUDEP and near-SUDEP cases [16,113]. The occurrence of post-convulsive central apnea is not associated with ictal central apnea and is likely driven by brainstem mechanisms involving the periaqueductal gray [113]. Furthermore, postictal posturing predicts a six-fold increased risk of post convulsive central apnea [54]. As more becomes understood, these features may represent possible biomarkers for SUDEP (Table 5).

## 6. Prevention

Minimizing SUDEP risk factors such as nocturnal seizures and GTCS frequency is the mainstay approach to mitigating SUDEP (Table 6). Prescribing a higher evening dose of ASM for nocturnal and early morning seizures (differential dosing) has been demonstrated to reduce seizure frequency and improve the rates of seizure freedom without adverse effects [114]. Because polytherapy is not associated with SUDEP risk when GTCS frequency is taken into consideration [80] and epilepsy surgery appears to decrease mortality in patients that achieve good seizure control [86], it appears that treating the seizures is an important component of mitigating SUDEP risk.

DORAs have been shown in preclinical studies to mitigate respiratory pathophysiology and sleep disturbance associated with SUDEP [100,106], but its utility for SUDEP mitigation in the clinical setting has yet to be assessed.

Adenosine augmentation therapies have been explored for epilepsy management [115]. The astroglial expression of adenosine kinase (ADK) is elevated in the setting of epilepsy [115]. Adenosine kinase catalyzes the breakdown of adenosine. Elevated ADK leads to reduced adenosine levels, reduced seizure threshold, and increased DNA methylation [115]. ADK inhibitors have been reported to potentiate seizure-related adenosine surges [116,117,118,119]. 5-iodotubercidin is a global ADK inhibitor that effectively reduces seizure frequency in a mouse model of temporal lobe epilepsy [120]. However, systemic ADK inhibition can cause liver toxicity [121] and cognitive issues [122]. Targeting specific ADK isoforms involved in epileptogenesis may be more favorable. Gene therapy that aims to knock down ADK expression has shown promise in preclinical models for reducing seizures [123]. Beyond ADK expression, adenosine-A2A receptor signaling has also been shown to play a role in mouse models of SUDEP. Adenosine-A2A receptor levels in the nucleus tractus solitarius were observed in epileptic mice, and an adenosine-A2A receptor antagonist significantly reduced SUDEP events in these mice [124]. While decreasing seizure frequency in people with epilepsy is an important theme in SUDEP prevention, it is important to keep in mind that excess adenosine in the postictal period can contribute to respiratory depression and the cascade of events that lead to SUDEP [122]. Thus, the goal of adenosine augmentation as it relates to SUDEP prevention should be to restore equilibrium, rather than to boost the acute seizure-induced adenosine response.

Serotonergic drug targets also have been gaining traction in recent years [125]. Fenfluramine is a serotonin 1D, 2A, 2C receptor agonist approved for the treatment of Dravet syndrome [126]. Fenfluramine decreases mortality in people with Dravet syndrome from 9.32 per 1000 person-years to 1.7 per 1000 [126]. SSRIs have been postulated to have utility in improving respiratory pathophysiology in the setting of SUDEP but have yet to demonstrate robust clinical evidence. Preclinical studies have shown that fluoxetine reverses respiratory arrest in a mouse model of epilepsy [127]. Clinically, SSRIs have been associated with reduced severity of ictal hypoxemia only in the setting of refractory focal epilepsy without generalization [63].

Iron chelators may play a neuroprotective role in preventing iron accumulation and minimizing ferroptosis in the context of epilepsy and SUDEP [72,73,74]. This has been explored in animal models but not clinically yet.

Vagus nerve stimulation (VNS) involves implanting a device that stimulates the vagus nerve with the goal of reducing seizure frequency [128]. SUDEP risk decreases in the 10 years following VNS implantation [129]. This is likely related to decreases in seizure frequency, but natural long-term changes in SUDEP incidence and autonomic impact of VNS may also play a role. The impact of VNS on autonomic function, specifically HRV, is controversial [130,131].

Responsive neurostimulation (RNS) involves implanting a device that monitors seizures and stimulates the brain to disrupt seizure activity [132]. SUDEP incidence has been reported as 2 per 1000 person-stimulation-years among patients treated with RNS, which is favorable compared to the control group [133].

Preclinical models have shown that the electrical stimulation of periaqueductal gray may compensate for respiratory mechanisms underlying SUDEP [134].

Diaphragmatic pacing is a surgical method of ventilating patients in a setting where endogenous respiration is inadequate, such as spinal cord injury or sleep apnea [135]. Preclinical studies in mice have shown that diaphragmatic pacing can prevent seizure-induced respiratory arrest [135,136].

As many cases of SUDEP are associated with prone positioning, implicating an element of asphyxiation, using an anti-asphyxia pillow may be of consideration [137].

Nighttime surveillance via motion-detecting seizure alarms may help trigger early intervention, which can mitigate fatal outcomes [16]. Wearable devices are also being developed to detect seizure [138]. There are, however, several cases of non-seizure-related SUDEP that suggest a role for apnea alarms as well [17].

## 7. Counseling

Counseling for caregivers and patients is important as it relates to SUDEP. The majority of pediatric neurologists provide information about SUDEP to only a select, high-risk group of children with epilepsy [139]. Parents of children with epilepsy generally do express the desire to have the conversation with their pediatric neurologist. There does exist a gap in education about SUDEP, with contributing factors including time constraint, cultural barriers, education resources, and physician perception of what patients want. Although receiving the information for the first time can be shocking, education about SUDEP does not create any long-term negative impact [139].

Counseling regarding SUDEP can involve a presentation of what SUDEP is, identifiable risk factors, and ways to potentially mitigate those risks. Cardiopulmonary resuscitation training may be discussed in conjunction with risk mitigation as well. Counseling should also include answering any questions that patients and caregivers might have and addressing any misconceptions.

## 8. Future Directions

In conclusion, SUDEP is the leading cause of epilepsy-related death in adults and children with epilepsy and should be investigated further with larger epidemiological studies to mitigate risk. Though the underlying mechanism is not yet fully understood, well-documented cases of SUDEP suggest that a GTCS-induced, centrally mediated change in cardiorespiratory function leads to terminal apnea and cardiac arrest [16]. The increased incidence of SUDEP during sleep [16] and the frequent sleep comorbidities [97] identified in epilepsy patients supports the notion that the role of sleep in SUDEP is an important avenue of further exploration. Serotonergic neurotransmission is also important in autonomic pathways [125] and may represent novel drug targets for SUDEP mitigation. In addition, further assessment of DORAs in SUDEP risk mitigation is warranted as a potential drug target.

## Figures and Tables

**Table 1:** Unified sudden unexpected death in epilepsy (SUDEP) definition and classification. Adapted from Box 3 of “Unifying the Definitions of Sudden Unexpected Death in Epilepsy” [4].

1. Definite SUDEP: ^a^ sudden, unexpected, witnessed or unwitnessed, nontraumatic, and non-drowning death, occurring in benign circumstances, in an individual with epilepsy, with or without evidence of a seizure and excluding documented status epilepticus (seizure duration ≥30 min or seizures without recovery in between), in which post-mortem examinations do not reveal a cause of death. 1a. Definite SUDEP Plus:^a^ satisfying the definition of Definite SUDEP, if a concomitant condition other than epilepsy is identified before or after death, if the death may have been due to the combined effect of both conditions, and if autopsy or direct observations/recordings of the terminal event did not prove the concomitant condition to be the cause of death. 2. Probable SUDEP/Probable SUDEP Plus: ^a^ same as Definite SUDEP but without an autopsy. The victim should have died unexpectedly while in a reasonable state of health, during normal activities, and in benign circumstances, without a known structural cause of death. 3. Possible SUDEP: ^a^ a competing cause of death is present. 4. Near-SUDEP/Near-SUDEP Plus: a patient with epilepsy survives resuscitation for more than 1 h after a cardiorespiratory arrest that has no structural cause identified after investigation. 5. Not SUDEP: a clear cause of death is known. 6. Unclassified: incomplete information available; not possible to classify.

^a^ If a death is witnessed, an arbitrary cutoff of death within 1 h from acute collapse is suggested.

**Table 2:** Mechanisms of sudden unexpected death in epilepsy (SUDEP).

Respiratory Central apnea Obstructive events Desaturation Hypercapnia with acidosis Bradypnea or tachypnea
Cardiac Heart rate variability Bradycardia Sinus arrest QT interval changes Atrial fibrillation
Postictal Generalized Electroencephalogram Suppression
Neurotransmitters Adenosine Serotonin
Ferroptosis

**Table 3:** Risk factors for sudden unexpected death in epilepsy (SUDEP).

Factor	Effect
Nocturnal seizures	The presence of nocturnal seizures is associated with increased risk of SUDEP.
Epilepsy onset	Early onset of epilepsy during pediatric age is associated with increased risk of SUDEP.
Seizure frequency	More frequent nocturnal generalized tonic clonic seizures are associated with increased risk of SUDEP.
Postictal generalized electroencephalogram suppression	Prolonged postictal generalized electroencephalogram suppression is associated with increased risk of SUDEP.
Genetics	Certain channelopathies and other genetic syndromes are associated with increased risk of SUDEP.

**Table 4:** Genetic conditions associated with sudden unexpected death in epilepsy (SUDEP).

Sodium channel mutations	SNCA1 SCN5A
Potassium channel mutations	KCNQ1 KCNH2
Developmental disorders	15q duplication syndrome CDKL5 deficiency disorder PCDH19-related epilepsy Ring chromosome 20 syndrome

**Table 5:** Possible biomarkers for sudden unexpected death in epilepsy (SUDEP).

Post-convulsive central apnea Heart rate variability Fragmented sleep Cardiac arrhythmias Postictal generalized electroencephalogram suppression

**Table 6:** Prevention of sudden unexpected death in epilepsy (SUDEP).

Decrease seizure burden Antiseizure medication compliance Avoidance of sleep deprivation and alcohol Vagus nerve stimulation Responsive neurostimulation Epilepsy surgery
Nighttime surveillance Motion-detecting seizure alarms Apnea alarms
Decrease respiratory risk factors Anti-asphyxia pillow Treat underlying sleep apnea

## Abbreviations

ADK	adenosine kinase
ASM	antiseizure medication
DORA	dual orexin receptor antagonist
EEG	electroencephalogram
GTCS	generalized tonic clonic seizure
HRV	heart rate variability
LQTS	long QT syndrome
NREM	non-rapid eye movement
PGES	postictal generalized electroencephalogram suppression
REM	rapid eye movement
rMSSD	root mean square difference
RNS	responsive neurostimulation
SCD	sudden cardiac death
SIDS	sudden infant death syndrome
SSRI	selective serotonin reuptake inhibitor
SUDEP	sudden unexpected death in epilepsy
VNS	vagus nerve stimulation
